# Bare-metal stent thrombosis two decades after stenting

**DOI:** 10.5830/CVJA-2015-034

**Published:** 2015

**Authors:** Aynur Acibuca, Demet Menekse Gerede, Veysel Kutay Vurgun

**Affiliations:** Department of Cardiology, Ankara University School of Medicine, Ankara, Turkey; Department of Cardiology, Ankara University School of Medicine, Ankara, Turkey; Department of Cardiology, Ankara University School of Medicine, Ankara, Turkey

**Keywords:** bare-metal stent, very late stent thrombosis, acute coronary syndrome, antiplatelet therapy

## Abstract

Very late bare-metal stent (BMS) thrombosis is unusual in clinical practice. To the best of our knowledge, the latest that the thrombosis of a BMS has been reported is 14 years after implantation. Here, we describe a case of BMS thrombosis that occurred two decades after stenting. A 68-year-old male patient was admitted with acute anterior myocardial infarction. This patient had a history of BMS implantation in the left anterior descending coronary artery (LAD) 20 years previously. Immediate coronary angiography demonstrated acute thrombotic occlusion of the stent in the LAD. With this case, we are recording the latest reported incidence of BMS thrombosis after implantation.

## Abstract

Stent thrombosis is a rare but serious complication that can lead to death or myocardial infarction (MI). Premature cessation of dual antiplatelet therapy is the most important risk factor.

Stent thrombosis is classified according to the ARC (academic research consortium) criteria as definite, probable or possible.^1^ Stent thrombosis can occur acutely (within 24 hours), sub-acutely (within 30 days), or as late as one year (late) or more (very late) after stent placement.

Both randomised trials and observational study data have demonstrated that the cumulative rate of stent thrombosis is similar for bare-metal and first-generation drug-eluting stents for up to five years.^2^,^3^ There may be a slight predominance of bare-metal stent (BMS) thrombosis between 30 days and one year, with a slight preponderance of drug-eluting stent (DES) thrombosis beyond one year.^4^

Here, we report a case of very late BMS thrombosis that presented as anterior myocardial infarction 20 years after stent implantation for left anterior descending coronary artery (LAD) disease.

## Case report

A 68-year-old non-diabetic, normotensive, ex-smoking male patient was admitted to our coronary care unit complaining of chest pain of one hour onset, which was unresponsive to nitroglycerin. This patient’s history included a bare-metal stent implantation for a proximal LAD lesion 20 years previously. He had been discharged on acetyl salicylic acid; however, one month later, he discontinued antiplatelet medication and stopped attending his control visits.

**Figure 1.**
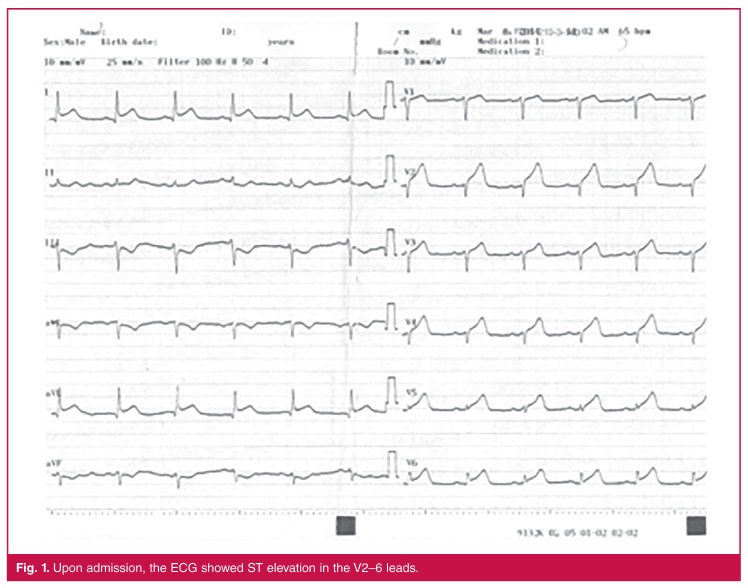
Upon admission, the ECG showed ST elevation in the V2–6 leads.

**Figure 2.**
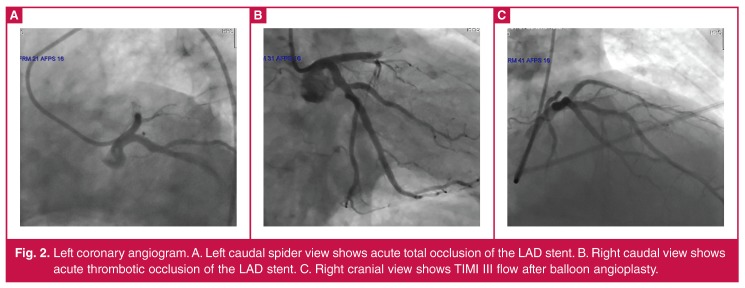
Left coronary angiogram. A. Left caudal spider view shows acute total occlusion of the LAD stent. B. Right caudal view shows acute thrombotic occlusion of the LAD stent. C. Right cranial view shows TIMI III flow after balloon angioplasty.

The electrocardiogram showed acute anterior myocardial infarction (Fig. 1), and an immediate coronary angiography showed total occlusion of the implanted stent in the proximal LAD (Figs 2A, B). Balloon angioplasty was therefore performed (Alvimedica balloon, 3 × 15 mm). TIMI 3 (thrombolysis in myocardial infarction) coronary flow was achieved (Fig. 2C); however, there appeared to be a need to perform percutaneous transluminal coronary angioplasty (PTCA) for the diagonal coronary artery branch (Advancer Hp balloon, 2 × 15 mm). Plaques were found on the right and circumflex coronary arteries. Following the administration of a bolus, tirofiban infusion was initiated post-procedurally and was continued for 24 hours.

During the follow-up period, ST-segment resolution was achieved. Transthoracic echocardiography showed modest impairment of the left ventricular systolic function with a global ejection fraction of 52%. On the sixth day after admission, this patient was discharged on dual antiplatelet therapy.

## Discussion

While very late stent thrombosis may be expected with the use of DES, it is rare with the use of BMS. A large, retrospective study reported that the cumulative incidence of stent thrombosis after BMS implantation was 1.3% at five years, and 2.0% at 10 years.^5^

The expected risk factors related to very late stent thrombosis are delayed neo-intimal proliferation and ongoing vessel inflammation.^6^ This is unexpected for BMS because stent endothelialisation is considered to be complete four weeks after the intervention. There are some case reports in the literature of very late thrombosis of BMS presenting with acute coronary syndrome occurring up to 14 years after implantation.^7^-^9^ However, many of them have no established cause.^8^,^9^

We believe this case of acute MI was caused by very late stent thrombosis, for a number of reasons. First, the patient was asymptomatic until this point; he had no angina or anginaequivalent symptoms. Second, a guide wire was easily passed through the occluded stent. Finally, balloon angioplasty was enough to obtain TIMI 3 coronary flow; there was no need for stent re-implantation.

The pathophysiology of very late BMS thrombosis is poorly defined. Independent predictors of stent thrombosis are premature discontinuation of antiplatelet therapy, renal failure, bifurcation lesions, diabetes, and lower ejection fraction.^10^ There are other presumed causes of BMS thrombosis, such as brachytherapy, exercise-induced procoagulant state, underdeployment of stents, malignancy, greater stent length, and small stent size.^11^

With the exception of the early discontinuation of antiplatelet therapy, our patient had none of these predisposing conditions. Interestingly, stent thrombosis did not occur immediately after the discontinuation of acetyl salicylic acid; it appeared after 20 years.

## Conclusion

Our report demonstrated evidence of the latest reported case of stent thrombosis with the use of BMS in the literature. Discontinuation or irregular use of antiplatelet agents can cause acute stent thrombosis, even decades after BMS implantation. This case emphasises the significance of lifelong antiplatelet therapy after stenting.
